# Bone marrow concentrate injections for the treatment of osteoarthritis: evidence from preclinical findings to the clinical application

**DOI:** 10.1007/s00264-020-04703-w

**Published:** 2020-07-13

**Authors:** Carola Cavallo, Angelo Boffa, Luca Andriolo, Simone Silva, Brunella Grigolo, Stefano Zaffagnini, Giuseppe Filardo

**Affiliations:** 1 Laboratorio RAMSES, IRCCS Istituto Ortopedico Rizzoli, Via di Barbiano, 1/10, 40136 Bologna, Italy; 2 Clinica Ortopedica e Traumatologica II, IRCCS Istituto Ortopedico Rizzoli, Via Pupilli, 1, 40136 Bologna, Italy; 3 Alma Mater Studiorum - Università di Bologna, Via Zamboni, 33, 40126 Bologna, Italy; 4 Applied and Translational Research (ATR) Center, IRCCS Istituto Ortopedico Rizzoli, Via di Barbiano, 1/10, Bologna, Italy

**Keywords:** Bone marrow concentrate, BMAC, Injective, Intra-articular, Osteoarthritis, Cartilage

## Abstract

**Purpose:**

To investigate the available literature on the use of bone marrow aspirate concentrate (BMAC) and summarize the current evidence supporting its potential for the injective treatment of joints affected by osteoarthritis (OA).

**Methods:**

A systematic literature search was conducted on three electronic databases (PubMed, Embase, and Cochrane Library) in April 2020, using the following string: “((bone marrow concentrate) OR (BMC) OR (bone marrow aspirate concentrate) OR (BMAC)) AND (osteoarthritis)”, and inclusion criteria: clinical and preclinical (animal) studies of any level of evidence, written in English language, and evaluating the intra-articular or subchondral use of BMAC for the injective treatment of OA joints.

**Results:**

The publication trend remarkably increased over time. A total of 22 studies were included in the qualitative data synthesis: four preclinical studies and 18 clinical studies, for a total number of 4626 patients. Safety was documented by all studies, with a low number of adverse events. An overall improvement in pain and function was documented in most of the studies, but the clinical studies present significant heterogeneity, few patients, short-term follow-up, and overall poor methodology.

**Conclusion:**

There is a growing interest in the field of BMAC injections for the treatment of OA, with promising results in preclinical and clinical studies in terms of safety and effectiveness. Nevertheless, the current knowledge is still preliminary. Preclinical research is still needed to optimize BMAC use, as well as high-level large controlled trials to better understand the real potential of BMAC injections for the treatment of patients affected by OA.

## Introduction

Osteoarthritis (OA) is a degenerative disease characterized by progressive deterioration and loss of articular cartilage with concomitant structural and functional changes in the entire joint [1]. Its incidence and prevalence are rising, in particular among older adults in developed countries, likely related to the aging of the population and increasing obesity [2–4]. Clinical features of OA are mostly characterized by signs and symptoms of inflammation, including pain, effusions, stiffness, and loss of mobility, often associated with significant functional impairment [5]. Current management strategies include weight loss, physical treatments, oral medications, such as non-steroidal anti-inflammatory drugs and acetaminophen, and intra-articular injection therapies with corticosteroids and hyaluronic acid (HA) [6]. Nevertheless, these treatment options mainly provide symptom relief rather than disease-modifying changes to the intra-articular environment [7].

The use of orthobiologics is gaining increasing interest due to the availability of new promising products to address OA. Among these, platelet concentrates are now considered a suitable option to treat OA, with results going beyond the mere placebo effects expected for injective treatments and higher than those provided by other traditional products, although with still suboptimal outcomes [8, 9]. More recently, mesenchymal stromal cells (MSCs) have been proposed as a promising alternative for OA treatment thanks to their structural contribution to tissue repair and even more their immunomodulatory and anti-inflammatory actions, through direct cell-to-cell interaction or secretion of bioactive factors [10, 11]. MSCs can be easily isolated from various tissues, such as bone marrow, adipose tissue, synovial membrane, peripheral blood, and skin [12]. Bone marrow MSCs (BMSCs) were the first type of MSCs to be identified and, due to the ease of collection, bone marrow currently represents a commonly used source of MSCs [13]. In particular, this type of MSCs has been either applied as a cell suspension after being expanded by culture or used as a simple bone marrow concentrate (BMC), thanks to their relative abundance [14]. Considering the strict regulations and the problems related to cell manipulation and expansion, cultured BMSCs have been widely explored in the preclinical setting, but their use is extremely limited in clinical practice, both in Europe and in the USA [15–19]. Conversely, the minimal cell manipulation approach, allowing to obtain a bone marrow aspirate concentrate (BMAC) directly on site in a one-step treatment, has been widely utilized in the clinical practice for the treatment of cartilage lesions first and, more recently, has been proposed as a promising injective approach to treat degenerative orthopaedic conditions [20, 21]. Nevertheless, the real potential of BMAC as intra-articular OA treatment remains controversial [22].

The aim of this systematic review was to examine the available literature on the use of BMAC, evaluating preclinical and clinical studies, in order to summarize the current evidence supporting its potential for the injective treatment of joints affected by OA. The hypothesis of the current study was that the available preclinical studies support the rationale for the injective use of BMAC, and that an increasing number of clinical studies reports safety and effectiveness of this biological approach.

## Material and methods

A systematic review of the literature was performed on the use of BMAC as injective treatment for joints affected by OA. A literature search was conducted on three electronic databases (PubMed, Embase, and Cochrane Library) on April 20th, 2020, with no time limitation and without any filters, using the following string: “((bone marrow concentrate) OR (BMC) OR (bone marrow aspirate concentrate) OR (BMAC)) AND (osteoarthritis)”. According to the Preferred Reporting Items for Systematic Reviews and Meta-Analysis (PRISMA) and Cochrane guidelines [23], the article selection and data extraction process were conducted separately by two authors (AB and SS). The initial title and abstract screenings were made using the following inclusion criteria: clinical and preclinical (animal) studies of any level of evidence, written in English language, and evaluating the intra-articular or subchondral use of BMAC for the injective treatment of joints with OA. Exclusion criteria consisted of articles written in other languages, literature reviews, basic science in vitro articles, case reports, congress abstracts, studies on joint diseases different from OA, studies on other BMAC applications (e.g., use as augmentation to other surgical techniques), and studies dealing only with expanded or otherwise manipulated MSCs. In the second step, the full texts of the selected articles were screened, with further exclusion according to the previously described criteria. Additionally, all references from the selected papers and previously published relevant reviews were also screened. Two investigators independently reviewed each article (AB and SS), and any discrepancies between them were resolved by discussion and consensus. For the included studies, relevant data were extracted from article texts, tables, and figures, and then summarized and analyzed according to the purpose of the present work. In particular, the following data were collected for preclinical studies: year of publication, animals evaluated, joint involved, OA model, types of treatment, BMAC harvest site and characteristics, follow-up length, evaluation methods, and results. For clinical studies, the following data were collected: year of publication, study design, joint involved, treatment type and schedule, BMAC manufacturing and characteristics, number of evaluated subjects, subject’s characteristics, follow-up length, main results, and adverse events.

The effectiveness of BMAC injective therapy was evaluated by summarizing the reported benefits, while the safety of the procedures was evaluated identifying the reported side effects. To assess the methodological quality of the included clinical studies, the subscales of a Coleman Methodology Score, modified by Kon et al. [24], were determined for each study by two separate authors (AB and SS). In case of disagreement between the two authors, divergences were discussed and a consensus was reached.

## Results

**Fig. 1:**
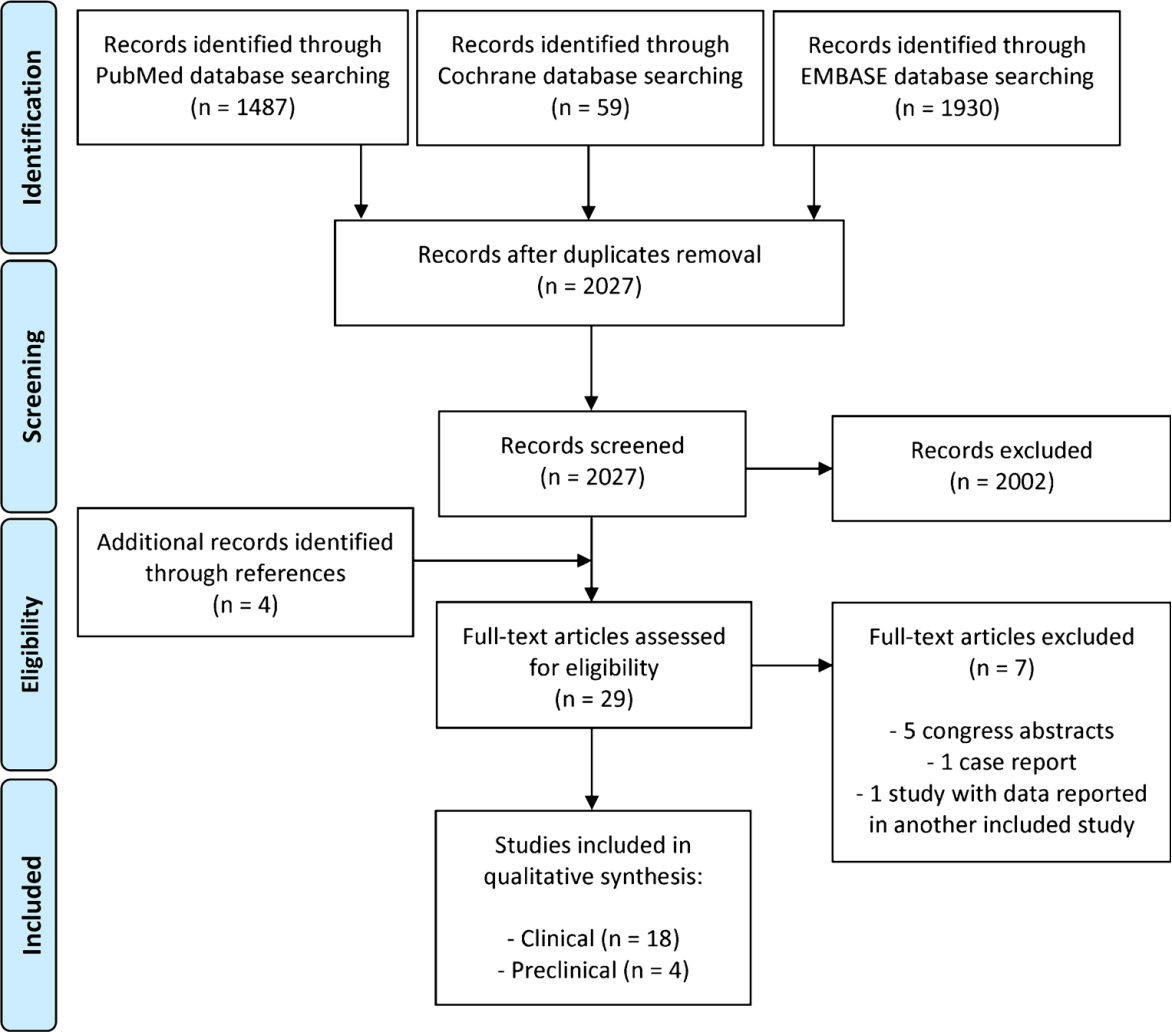
Flowchart showing the systematic review selection process

**Fig. 2:**
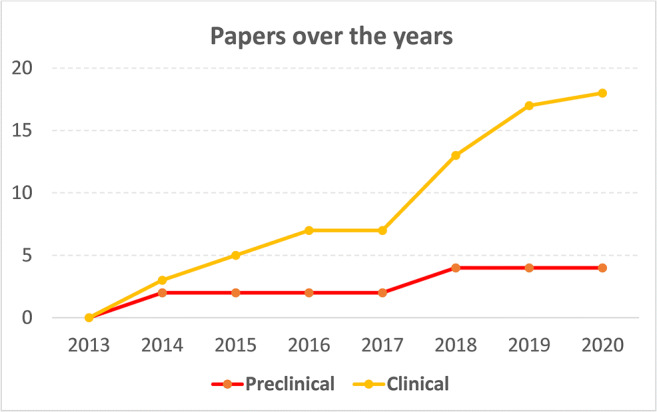
Cumulative number of preclinical and clinical studies on BMAC injective OA treatment

The flowchart of the article selection process is reported in Fig. 1. After duplicates were removed, the initial search identified 2027 records, whose abstracts were screened and selected according to the inclusion/exclusion criteria. Four articles were identified through the reference lists, which gave a total of 29 articles assessed for eligibility. However, five studies were excluded being congress abstracts, one study was excluded because it was a case report [25], and one study was excluded because it provided the same data of another included study [26]. Thus, a total of 22 studies were included in the qualitative data synthesis: 4 preclinical studies [27–30] and 18 clinical studies [31–48]. Since the first reports in 2014, the publication trend remarkably increased over time, especially for the clinical studies, with over 50% of the articles published from 2018 (Fig. 2).

### Preclinical studies

The potential of BMAC for the injective OA treatment has been tested in 4 animal studies (Table 1). All of them were comparative studies investigating the results of BMAC in knee OA models against placebo (saline), HA, platelet-rich plasma (PRP), and cultured BMSCs. Knee OA was induced by anterior cruciate ligament transection in all four studies, also associated with meniscectomy in two studies. Bone marrow was harvested from the iliac crest in three studies and from the proximal tibia in one study. The used animals included rabbit (two studies), sheep, and goat (one study each), which were sacrificed from six to 20 weeks after the intra-articular injections, and then evaluated through macroscopic and radiologic evaluation, histological examination, and immunohistochemical analysis.

**Table 1:** Preclinical studies

Author and year	Animal and OA model	Joint	Treatment	Harvest site	BMAC characteristics	Follow-up and evaluation	Results
Desando G et al. 2018 [27]	Rabbit (48 knees) OA induced by ACL transection	Knee	12 BMAC vs. 12 cultured BMSCs vs. 12 BMAC + HA vs. 12 cultured BMMCs + HA Single IA injection	Iliac crest	Centrifugation procedure at 400*g* for 10 min at 20 °C 2 × 10^6^ cells/ml	8 weeks Cell biodistribution, histologic, immunohistochemical, and synovial fluid analysis	BMAC showed effectiveness for the injective OA treatment, with similar results than BMSCs and better results for the association of BMAC + HA.
Wang Z et al. 2018 [28]	Goat (24 knees) OA induced by ACL transection and meniscectomy	Knee	6 BMAC vs. 6 saline vs. 6 PRP Three IA injections (once every 4 weeks)	Iliac crest	Two-step centrifugation procedure 5 ml with 1190.3 × 10^6^ cells/ml	6 weeks Macroscopic, histologic, immunohistochemical, and synovial fluid analysis	Higher cartilage protection in the BMAC group than both PRP and saline groups.
Song F et al. 2014 [29]	Sheep (18 knees) OA induced by ACL transection and meniscectomy	Knee	6 BMSCs vs. 6 saline vs. 6 cultured MSCs Single IA injection	Iliac crest	BMSCs were isolated by density gradient centrifugation, and were resuspended in PBS 5 ml with 0.9 × 10^6^ cells/ml	8 weeks Macroscopic, histopathologic, ELISA and proteoglycan assay, RT-PCR analysis of cartilage	BMSCs and cultured MSCs provided both good results in terms of macroscopic and histologic findings, GAG protection, cytokines, and cartilage biomarkers changes.
Singh A et al. 2014 [30]	Rabbit (20 knees) OA induced by ACL transection	Knee	10 BMSCs vs. 10 saline Single IA injection	Proximal tibia	BMSCs were isolated by density gradient following Pittenger’s method 1 ml with 1 × 10^6^ cells/ml	20 weeks Macroscopic, radiologic, and histopathologic analysis	BMSCs provided significant histopathological and radiological improvement compared with control group.

*ACL* anterior cruciate ligament; *BMAC* bone marrow aspirate concentrate; *BMSCs* bone marrow mesenchymal stromal cells; *ELISA* enzyme-linked immunosorbent assay; *HA* hyaluronic acid; *IA* intra-articular; *MSCs* mesenchymal stromal cells; *PBS* phosphate-buffered saline; *PRP* platelet-rich plasma; *RT-PCR* real-time polymerase chain reaction

The macroscopic evaluation of articular cartilage was described in three studies, all showing better results in animals treated with BMAC compared with control groups. Song et al. [29] and Singh et al. [30] reported less severe cartilage deterioration compared with saline. In addition, beside showing the benefits vs. saline, Wang et al. [28] documented in the BMAC group only focal and superficial cartilage erosion and mild osteophyte development with respect to the PRP group, where obvious extensive and full-thickness cartilage defects combined with marked osteophyte development were reported. Singh et al. [30] performed also a radiological evaluation, confirming the positive results by showing less severe signs of OA (including osteophyte formation, subchondral bone sclerosis, and articular surface irregularity) when isolated non-cultured BMSCs were injected compared with saline.

The histological analysis was performed in all studies, reporting better results in the BMAC group, with degenerative changes and articular cartilage erosion limited within the superficial layer, normal chondrocyte density, and abundant extracellular matrix preserved in the lower layers. Interestingly Desando et al. [27] in a rabbit model demonstrated similar results with respect to the use of cultured BMSCs and better histological findings in joints treated with the combination of BMAC and HA with respect to BMAC alone. Moreover, they underlined the cell homing pattern: MSCs preferentially migrated toward tissue areas showing OA features in the meniscus and cartilage, and near inflammatory zones in the synovial membrane, and the combination with HA contributed to boost cell migration toward articular cartilage. Desando et al. [27] also investigated immunohistochemical characteristics, showing a downregulation of type I collagen and TNF-α in articular cartilage after BMAC treatment. Finally, Song et al. [29] evaluated GAG content showing an increase in the proteoglycan concentration in the cellular treatment groups. Moreover, they analyzed the gene expression of Col2A1, Aggrecan, and MMP-13 underlining a significant increase in Col2A1 and Aggrecan, and a decrease in MMP-13 in both non-cultured BMSC and cultured MSC groups compared with the saline group.

### Clinical studies

Out of the 18 clinical articles found (Table 2), six were retrospective case series, five were retrospective comparative studies, four were randomized controlled trials (RCTs), and three were prospective case series. Eleven studies focused on knee OA [31–41], two focused on hip OA [42, 43], two focused on shoulder OA [44, 45], while the other three studies described several joints affected by OA [46–48]. Intra-articular BMAC injections were performed in 17 studies, while in one study [36], the injection was performed within the tibial and femoral subchondral bone of the knee. The most common injection schedule was the single injection (16 articles), while a four injection schedule was studied in two articles [37, 42]. In 11 studies, BMAC was the only product injected, while in the other seven studies, other products were combined including PRP, platelet lysate, or minimally processed adipose tissue. In the nine studies with control groups, the effectiveness of BMAC was compared with other injective treatments such as PRP, micro-fragmented adipose tissue, whole bone marrow, cultured MSCs, placebo, or other treatments such as exercise and total knee arthroplasty. The BMAC harvest site was the anterior-superior (ASIS) or postero-superior iliac spine (PSIS) (14 PSIS, 3 ASIS, and 1 ASIS or PSIS). The total number of subjects included and treated with BMAC injections was 4626, and the mean trial duration was 20 months, ranging from two months to 12 years of follow-up, with all but one study [36] lasting less than 2.5 years. The evaluation with the modified Coleman Methodology Score showed an overall poor methodology of the included studies, with an average score of 36.6 points out of 100 (range 23–60), as reported in Table 2.

**Table 2:** Clinical study characteristics

Author and year	Study design	Joint	Treatment	Harvest site	Bone marrow concentration and injected amount	Patient, *n* (M/F) Age, mean (SD or range)	F-up	Results and adverse events	mCMS
Anz AW et al. 2020 [31]	Unblinded RCT	Knee	BMAC vs. LR-PRP Single IA injection	PSIS	Dual-Spin protocol/disposable PureBMc - EmCyte (Fort Myers, FL, USA) 7 ml of BMAC	BMAC group 45 (27/18) 56 years (11) LR-PRP group 41 (22/19) 52 years (12)	12 m	All scores for both PRP and BMAC groups significantly improved up to 12 months, with no difference between PRP and BMAC at any time point. No adverse events were reported.	60
Mautner K et al. 2019 [32]	Retrospective comparative study	Knee	BMAC vs. MFAT Single IA injection	PSIS	EmCyte centrifuge (Fort Myers, FL, USA) 8 ml of BMAC	BMAC group 41 (24/17) 59 years (1) MFAT group 35 (12/23) 63 years (11)	22 m	There were significant improvements in pain and function with both treatments, without a significant difference between the two groups. No adverse events were reported.	35
Shapiro SA et al. 2019 [33]	Blinded RCT (self-controlled)	Knee (bilateral)	BMAC vs. saline Single IA injection	PSIS	Magellan Autologous Platelet Separator System (Arteriocyte, USA) 5 ml of BMAC + 10 ml of PPP BMAC product contained a median of 56% MNCs (150 × 10^6^ WBC, 80 × 10^6^MNC, 4.4 × 10^6^ hematopoietic stem cells, and 34,000 MSCs)	Bilateral OA patients 25 (7/18) 60 years (42–68)	12 m	No difference between BMAC- and saline-treated knees in patients with bilateral knee OA. No adverse events were encountered.	57
Subasi T et al. 2019 [34]	Prospective case series	Knee	BMAC Single IA injection (followed 1 month later by a PRP injection)	PSIS	MC kitTM (nFinders, Seoul, Korea) 4 ml of BMAC	3 (0/3) 58 years (10)	2 m	IA BMAC and PRP treatments may have positive effects on pain, functional status, and quality of life in patients with knee OA. No side effects or tractable pain were detected.	27
Centeno C et al. 2018 [35]	Unblinded RCT	Knee	BMAC + platelet products vs. exercise therapy (pre-injection of dextrose, post-injection of PRP, hydrocortisone, and doxycycline after 2–4 days) Single IA injection	PSIS	The aspirate was processed in a sterile ISO-7 class clean room 5–7 ml injectate solution (75% of BMAC)	BMAC group 26 54 years (9) Exercise group 22 57 years (8)	24 m	The use of BMAC with platelet products yielded better results than exercise therapy. No serious adverse events were identified. One patient reported a persistent popliteal fossa fluid accumulation, which was aspirated.	55
Hernigou P et al. 2018 [36]	Unblinded RCT	Knee	BMAC vs. TKA Single subchondral injection	ASIS	40 ml of BMAC in the subchondral medial and lateral femorotibial compartments. BMAC contains an average of 6500 MSCs/ml	30 (12/18) 28 years (18–41)	144 m	Subchondral BMAC was an effective procedure for treating young patients with knee OA following secondary osteonecrosis. During the postoperative period, the number of adverse events was higher in the TKA group.	67
Shaw B et al. 2018 [37]	Retrospective case series	Knee	BMAC 4 IA injections (1 every 2 weeks)	PSIS	The BMAC was spun in a centrifuge, and the upper portion without visible red cells was isolated from the centrifuged BMAC. 5 ml of BMAC + 1 ml of ropivacaine	15 (5/10) 68 years (8)	3 m	Outcomes at the final follow-up after the fourth treatment were statistically significant compared with outcomes at baseline. No serious adverse events were reported.	24
Themistocleous GS et al. 2018 [38]	Retrospective case series	Knee	BMAC Single IA injection	ASIS	Single-spin centrifugation technique (Hettich® Rotofix 32A centrifuge) 10 ml of BMAC	121 (35/86) 70 years (50–85)	11 m	BMAC was a safe and reliable procedure that resulted in clinical improvement in patients with grades 3 and 4 knee OA. There were no adverse events or complications.	41
Centeno C et al. 2015 [39]	Retrospective comparative study	Knee	BMAC (higher cell count) + platelet products vs. BMAC (lower cell count) + platelet products Single IA injection	PSIS	The aspirate was processed in a sterile ISO-7 class clean room. Higher cell count group BMAC: > 4 × 108 cells Lower cell count group BMAC: ≤ 4 × 108 cells	Higher cell count 224 54 years (13) Lower cell count 185 50 years (16)	12 m	Patients receiving a higher concentration of cells reported a better pain outcome in comparison with the lower dose group. No adverse events were reported.	28
Centeno C et al. 2014 [43]	Retrospective comparative study	Knee	BMAC + platelet products vs. BMAC + platelet products + adipose graft (pre-injection of dextrose) Single IA injection	PSIS	The aspirate was processed in a sterile ISO-7 class clean room. 1–3 ml BMAC injected intra-articularly and in painful or damaged structures	Group A 518 54 years (14) Group B 163 60 years (10)	12 m	Although improvements were statistically significant, the differences between the groups were not. No adverse events secondary to the procedure were reported.	23
Kim JD et al. 2014 [41]	Prospective case series	Knee	BMAC + adipose tissue Associated surgery in 16% of patients Single IA injection	ASIS or PSIS	SmartPReP2 BMAC2 kits (Harvest Technology, USA) 7 ml BMAC + 10 ml ADSCs	41 (17/24) 61 years (53–80)	12 m	BMAC injection significantly improved both knee pain and function in the patients with knee OA. No adverse events were reported.	44
Darrow M et al. 2018 [42]	Retrospective case series	Hip	BMAC 4 intra-articular injections (1 every 2 weeks)	PSIS	The BMAC was spun in a centrifuge, and the upper portion without visible red cells was isolated from the centrifuged BMAC. 5 ml of BMAC + 1 ml of ropivacaine	4 (3/1) 67 years (10)	4 m	All patients experienced decreased pain and improved functionality compared with baseline. No adverse events were reported.	23
Centeno C et al. 2014 [43]	Retrospective case series	Hip	BMAC + platelet products (pre-injection of dextrose) Single IA injection	PSIS	The aspirate was processed in a sterile ISO-7 class clean room. 1–4 ml of BMAC + 2 ml of PRP + PL	216 (124/92) 57 years (11)	9 m	BMAC injections for hip OA demonstrated encouraging results for improved outcomes with no significant complications. There were no severe or serious adverse events.	26
Darrow M et al. 2019 [44]	Retrospective comparative study	Shoulder	BMAC vs. WBM 1 or 2 IA injections	PSIS	The BMAC was spun in a centrifuge, and the upper portion without visible red cells was isolated from the centrifuged BMAC. 5 ml of BMAC + 1 ml of ropivacaine	32	8 m	Injective BMAC or WBM treatment in patients with shoulder OA significantly improved resting pain, active pain, and functionality score, with greater results in group with 2 injections. No adverse events were reported.	24
Centeno C et al. 2015 [45]	Retrospective case series	Shoulder	BMAC + platelet products (pre-injection of dextrose) Single IA injection	PSIS	The aspirate was processed in a sterile ISO-7 class clean room. 1–4 ml of BMAC + 1 ml of PRP + 1 ml of PLCell count: 3.85 × 10^8^	34 (27/7) 52 years (17)	11 m	The use of BMAC to treat shoulder OA was effective at improving pain and function. No serious adverse events were reported.	29
Rodriguez-Fontan F et al. 2018 [46]	Prospective case series	Knee Hip	BMAC Single IA injection	ASIS	BioCUE Platelet Concentration System (Zimmer Biomet, Warsaw) 12 ml of BMAC	19 (3/16) 58 years (13)	24 m	IA injections of BMAC for the treatment of early knee or hip OA were safe and demonstrated satisfactory results in 63.2% of patients. No serious adverse events were reported.	42
Centeno C et al. 2016 [47]	Retrospective comparative study	Knee Hip Ankle Shoulder	(A) BMAC + platelet products vs. (B) BMAC + platelet products + adipose graft vs. (C) cultured MSCs (pre-injection of dextrose) Single IA injection	PSIS	The aspirate was processed in a sterile ISO-7 class clean room 1–3 ml of BMAC containing 0.2–1.5 × 108 nucleated cells	A: 246 (134/113) 60 years (11) B: 1589 (964/626) 56 years (14) C: 535 (343/192) 53 years (13)	26 m	Adverse events were reported in 12.1% of the study population. Lowest rate of adverse events was among those patients receiving BMAC injections alone, compared with patients that received also adipose graft.	24
Sampson S et al. 2016 [48]	Retrospective case series	Knee Ankle Hip Shoulder	BMAC Single IA injection PRP injection after 8 weeks	PSIS	Thermo Scientific CL2 centrifuge (MA, USA) Larger joints: 5 ml of BMAC Smaller joints: 1–2 ml of BMAC	125 57 years (23–79)	5 m	IA injection of BMAC, followed by a PRP injection, provided short-term benefits in OA. No adverse events were reported.	29

*ASIS* anterior superior iliac spine; *BMAC* bone marrow aspirate concentrate; *BMMNCs* bone marrow mononuclear cells; *mCMS* modified Coleman Methodology Score; *IA* intra-articular; *LR* leukocyte rich; *m* months; *MFAT* micro-fragmented adipose tissue; *MNCs* mononuclear cells; *OA* osteoarthritis; *PL* platelet lysate; *PSIS* posterior superior iliac spine; *PRP* platelet-rich plasma; *RCT* randomized controlled trial; *SD* standard deviation; *TKA* total knee arthroplasty; *WBM* whole bone marrow

The main finding of the included studies was an overall improvement in pain and function in OA patients treated with BMAC injections, with similar results obtained for the different joints evaluated. However, the comparative studies were not able to prove superiority over the other intra-articular options and, in the only placebo-blinded RCT [33], BMAC did not show superiority over saline at 12 months of follow-up. On the other hand, intra-articular BMAC injections (combined with platelet products) demonstrated better results than exercise therapy in knee OA patients at 24 months of follow-up [35], and subchondral BMAC injections reported similar results compared with total knee arthroplasty in younger patients with knee OA secondary to corticosteroid-related osteonecrosis at an average of 12 years of follow-up [36].

Shaw et al. [37] underlined the importance of the injection schedule, suggesting that multiple BMAC injections can be more effective than a single injection, and reported additional benefit with each subsequent treatment both for knee and hip OA. In addition, Centeno et al. [39] suggested that patients receiving a BMAC injection with a higher concentration of cells reported a better pain outcome in comparison with the lower dose group. The role of OA severity was underlined in two studies [41, 43] showing better clinical results after intra-articular BMAC injections in patients with moderate OA compared with those with severe OA (Kellgren-Lawrence grade 4). Finally, the possibility to improve the results by combining different products was investigated in two comparative studies [40, 47], where the combination of BMAC and adipose tissue did not prove superiority with respect to BMAC alone.

Safety was documented by all studies, with no severe adverse events related to the injective procedures. The most common reported adverse effects were temporary pain or joint swelling during the first weeks after BMAC injection, followed by grinding, popping, and snapping sensations with specific movements. Centeno et al. [47] documented in a multi-center safety analysis a 12% rate of adverse events in a large group of OA patients treated with intra-articular injections of BMAC. In particular, patients receiving BMAC injections alone reported fewer adverse events with respect to patients treated with intra-articular combined injections (e.g., BMAC plus adipose tissue). The same group also reported in another study [35] one patient with a persistent popliteal fossa fluid accumulation after the injection procedure, which was aspirated and resolved. Finally, Hernigou et al. [36] reported a higher safety rate in patients treated with knee subchondral BMAC injections compared with patients who underwent total knee arthroplasty.

## Discussion

The main finding of this systematic review is that the available preclinical and clinical studies suggest safety and overall positive results of the injective treatment with BMAC in joints affected by OA. This research highlighted a growing number of clinical trials published in the last years. Nevertheless, the literature analysis also underlined the limits of this young field, with only a few preclinical studies supporting the rationale of BMAC injections, as well as an overall low quality of evidence of the clinical studies.

BMAC is increasingly used as injective treatment of OA, with the rationale relying on the transplantation of the entire bone marrow niche which contains MSCs, haematopoietic precursors, monocytes, and endothelial cells, as well as a great array of soluble factors [14, 49, 50]. BMSCs have the capacity to differentiate toward several lineages (i.e., chondrocytes, osteoblast, adipocytes) and to produce soluble factors, which may positively affect the joint homeostasis and eventually contribute to relief pain and to improve joint function [21, 51, 52]. In particular, MSCs possess immunomodulatory, anti-inflammatory, anti-apoptotic, proliferative, and chemoattractive functions, and can coordinate the differentiation process of functional tissue regeneration in host cells [53]. However, the amount of BMSCs in BMA is not elevated, with a study investigating CD34^−^, CD45^low^, and CD271^high^ positive cells underlying a low 0.04% rate [54]. Quantifying BMSCs has been historically based on the number of colony-forming units (CFU) that emerge from in vitro culture of BMA samples, with studies estimating between 109 and 664 CFU per milliliter of BMA [55]. BMAC only slightly increases the concentration of BMSCs, leading to a much lower number of BMSCs compared with what was administered in studies on the injection of expanded BMSCs, where a range from 5 × 10^6^ cell/ml to 24 × 10^6^ cell/ml [14] has been reported. Nevertheless, compared with cultured BMSCs, BMAC contains a high number of platelets rich in growth factors, cytokines, and chemokines, including transforming growth factor-beta (TGF-β), interleukin-1 receptor antagonist (IL-1ra), platelet-derived growth factor (PDGF), bone morphogenetic protein (BMP)-2 and -7, and vascular endothelial growth factor (VEGF). These growth factors are involved in several pathways crucial for cell maintenance and function, for differentiation, for extracellular matrix production, and for the regulation of cell catabolic/anabolic activity [56–58]. Accordingly, this combination of cells and bioactive proteins makes BMAC a unique product among the orthobiologics presently available, and may present the potential to alter the disease course and not just to decrease pain [59]. Beside this rationale, the increasing use of BMAC is also due to the severe restrictions and regulatory issues related to other strategies to exploit MSCs, which involve extended cell manipulation and in vitro cultivation. This, together with the risk of infection, contamination, or cell trans-differentiation [17–19], makes cultured MSCs use extremely limited in the clinical practice both in Europe and in the USA [15]. On the other hand, the use of BMAC is authorized by the US Food and Drug Administration (FDA) because it is considered “minimally manipulated,” although its use is foreseen only for a homologous purpose and without involving a combination of cells or tissues with another substance [15]. According to this regulatory window, several different products have been proposed in the clinical practice to produce BMAC.

BMAC is frequently obtained through density gradient centrifugation of BMA, usually collected from the posterior or anterior iliac crest, and rarely from the distal femoral or proximal tibial metaphysis, the latter option being proposed in particular for knee pathology treatment [60]. The harvest site may play an important role for the product obtained. In a recent study, Davies et al. [61] suggested that the pelvis was superior to femur and tibia in terms of the number of stem cells isolated, even though there was no significant difference in the phenotype of the cells isolated from different locations. Moreover, Pierini et al. [62] demonstrated a higher quality of BMA when it was collected from the posterior rather than anterior iliac crest in terms of number of colony-founding connective-tissue progenitors. Beside the anatomical site, other factors can influence the quality of BMAC. Some authors reported a variable stem cell quantity and quality depending on age, including an age-related reduction in the absolute number of MSCs within BMA and a decreased proliferative and differentiative capacity [63, 64]. Finally, the production process itself might influence the biological properties of BMAC. The collected BMA is usually centrifuged directly in the operating room, and nucleated cells (stem cells, monocytes, lymphocytes) and proteins (growth factors, cytokines, and chemokines) are concentrated by removing most of the red cells and plasma. Then, BMA is reduced in volume with a cell separator to obtain 3–8 ml of BMAC, and the final volume used will depend from the clinical application. Even though these steps are common to most of the procedures developed to obtain BMAC by BMA centrifugation, there are several commercial systems suitable for producing BMAC. The lack of standardization of the producing techniques (such as differences in BMA starting volumes, centrifuge devices, and many other methodological differences) leads to several biological products with different progenitor cells and platelet number, other than different concentrations of growth factors and cytokines [65, 66]. This aspect may hinder the possibility to compare literature results and to understand the real potential of BMAC as OA injective treatment.

Despite the many still controversial aspects, BMAC is technically easy and presents the advantages of overcoming the need for culture expansion, thus reducing the risk of infection and avoiding the risk of allogeneic diseases [67]. Thus, BMAC use is growing exponentially in the clinical practice for several orthopaedic procedures, including the treatment for OA [68, 69]. In a previous literature review, Di Matteo et al. underlined that currently BMAC, together with adipose tissue-derived concentrates, were the most common strategies to exploit MSC potential through minimal manipulation, both showing promising results for knee OA, but also an overall poor methodology [65]. Nevertheless, it is important to underline that, while a large literature is available for other types of BMAC applications, the clinical OA application is poorly justified by preclinical evidence on this specific treatment indication. Only few preclinical studies focused on the intra-articular injective use of BMAC for OA, while the majority of these studies focused on culture-expanded BMSCs or on surgical applications of BMAC, including the treatment of focal cartilage defects (surgical augmentation or scaffold-based applications) [14, 70, 71]. The few preclinical studies in the literature suggested that BMAC can affect OA progression in the animal models, but they also underlined its limited potential, and the possibility to further exploit it through different production protocols as well as the combination of injective carriers to positively affect cell migration and favor longer-lasting homeostatic and disease-modifying effects [51, 72].

BMAC has been studied mainly for the knee injective treatment, while only few studies focused on shoulder, hip, or ankle OA. Overall, most of these studies converged on the safety and benefit of BMAC for OA symptom management. Better results were found for BMAC injections against exercise therapy in 48 patients with symptomatic knee OA, where this injective treatment suggested a greater impact on patients’ knee function [35]. Moreover, BMAC injections were found to provide clinical results comparable with TKA in a population of 30 young patients affected by osteonecrosis-related OA but with lower adverse events, thus suggesting, with all the limits inherent of a small survey, the possibility to delay joint replacement through the application of a regenerative therapy [36]. However, it is important to underline that no comparative study reported a superiority of BMAC against other injective treatments and, more importantly, the only available placebo-controlled RCT was not able to show any difference between BMAC- and saline-treated knees in patients with bilateral knee OA. Injective treatments, especially the “regenerative” ones, present a high and long-lasting placebo effect [8], and high-level trials will have to prove that BMAC can exceed the mere placebo effect, to confirm the promising findings suggested by the published studies and justify its use in the clinical practice.

A new application has been recently proposed to further exploit the potential of BMAC by targeting the subchondral bone which is commonly affected by the OA processes [36]. This strategy is supported by histological studies underlying different potential patterns of OA initiation, including initial subchondral bone alterations which may progress to the destruction of the overlying articular surface [73, 74]. Accordingly, the subchondral bone is becoming more and more a target and subchondral injective therapies are gaining an increasing interest, ranging from bone substitutes to biological treatments [75]. In particular, some authors reported the safety and effectiveness of subchondral PRP injections for the treatment of severe knee and hip OA [76–78]. Sanchez et al. [79] suggested a superior clinical outcome at six and 12 months for the combination of subchondral and intra-articular PRP injections when compared with intra-articular injections alone. The same authors suggested the possibility of subchondral PRP injections to delay arthroplasty in severe knee OA [80]. Similarly, Vad et al. [81] showed an improvement in pain and function in patients with knee OA treated with subchondral injections of BMA as an alternative to total knee replacement when other minimally invasive therapies have failed. Still, the subchondral application of BMAC has currently a limited scientific support and, as per intra-articular injections, more high-level studies are needed to understand if this could represent a valid option among biological treatment strategies for OA.

This systematic review presents several limitations reflecting the limitations of the available studies. In fact, the overall level of evidence was low; only four RCTs with a small size are available, while the other studies were retrospective comparative studies or case series. Another significant aspect is the absence of a proper control group comparison, with only one placebo-controlled trial and overall few and heterogeneous comparative studies performed. Many differences were documented among the included studies in terms of not standardized treatment protocols and different outcome measures. To this regard, many authors evaluated BMAC used with other orthobiologics, such as PRP or adipose tissue, hindering the possibility to isolate and assess the efficacy of BMAC as monotherapy. Moreover, BMAC preparation method, characteristics, and application modalities (MSC count, injected amount, injection schedule) were often different or not even reported by the authors. In addition, most of the studies used different scoring systems at different follow-ups, making it difficult to compare and merge results, thus impairing the possibility to perform a meta-analysis and to draw clear conclusions. Finally, the relatively short follow-up in most of these studies leaves concerns regarding the durability of the treatment results with BMAC for OA. Albeit being considered minimally invasive, this treatment still requires a surgical approach and results should prove significant and long-lasting to justify its use vs. less invasive homeostatic treatments [69].

In conclusion, this systematic review suggested a growing interest in the field of BMAC injections for the treatment of OA, with promising results in preclinical and clinical studies in terms of safety and effectiveness. Nevertheless, the current knowledge is still preliminary, the literature includes scarce preclinical evidence supporting BMAC rationale for this application, and clinical studies present significant heterogeneity, few patients, short-term follow-up, and overall poor methodology. Many aspects remain to be clarified to optimize the potential of BMAC, including methods of harvest, centrifugation, timing of injections, and application method, and to provide a standardized method targeted for OA treatment. Preclinical research is still needed to optimize BMAC use, as well as high-level large controlled trials to better understand the real potential of BMAC injections for the treatment of patients affected by OA.

## Data Availability

Not applicable.
